# Integrative analysis of whole-transcriptome sequencing reveals a ceRNA regulatory network centered on P4ha1 in liver cirrhosis treated with Pien Tze Huang

**DOI:** 10.17179/excli2026-9413

**Published:** 2026-07-13

**Authors:** Yunxiao Lin, Fan Yang, Yingtian Zhang, Xiaoqin Zhang, Xiangyi Li, Zhiliang Chen, Xianglong Zhao, Yongzhi Wang, Hao Wu, Cong Huai, Qiange Xiao, Wei Bao, Minglei Yang, Ruoyu Chen, Zhongyu Cao, Jinhang Zhu, Zekun Yu, Zexiu Zhang, Shengying Qin

**Affiliations:** 1 Bio-X Institutes, Key Laboratory for the Genetics of Developmental and Neuropsychiatric Disorders (Ministry of Education), Shanghai Jiao Tong University, Shanghai, 200030, China; 2 Fujian Provincial Key Laboratory of Pien Tze Huang Natural Medicine Research and Development, Zhangzhou Pien Tze Huang Pharmaceutical Co., Ltd, Zhangzhou, 363000, China; 3 Department of Pathology, The First Affiliated Hospital of Zhengzhou University, Zhengzhou, 450052, China

**Keywords:** cirrhosis, drug treatment, biomarkers, transcriptomics, Pien Tze Huang

## Abstract

Liver cirrhosis represents a terminal stage of chronic liver disease with limited pharmacological options. Pien Tze Huang (PTH), a traditional Chinese medicine with established hepatoprotective properties, has shown therapeutic potential in liver fibrosis, yet its molecular mechanisms in cirrhosis remain poorly understood. Here, we investigated the anti-cirrhotic effects of PTH and underlying mechanisms through whole-transcriptome sequencing in a carbon tetrachloride (CCl_4_)-induced mouse model of cirrhosis. PTH treatment significantly attenuated liver injury and other cirrhotic phenotypes, as evidenced by reduced serum transaminases and improved histological features. Transcriptomic analysis identified 1,518 genes dysregulated in cirrhosis and reversed by PTH treatment, with functional enrichment implicating inflammatory and fibrotic pathways including NF-κB, TNF, and IL-17 signaling. Connectivity Map analysis revealed similarity between PTH-induced transcriptional signatures and those of statins and beta-blockers, drugs currently evaluated in cirrhosis clinical trials. Through integrative construction of competing endogenous RNA (ceRNA) networks, we identified a core regulatory module comprising 6 miRNAs, 8 mRNAs, and 27 circRNAs with reversed expression patterns following PTH treatment. Notably, the liver-specific miR-122-5p exhibited the highest network connectivity, targeting *P4ha1*, a rate-limiting enzyme in collagen biosynthesis. Human proteome data confirmed liver-enriched expression of *P4HA1*, which is a validated therapeutic target of Lufironil, a Phase II clinical trial drug for cirrhosis. Our findings suggest that PTH ameliorates cirrhosis through multi-pathway regulation of a *P4ha1*-centered ceRNA network. Functional validation in hepatocytes confirmed that PTH modulates this network via the miR-122-5p/*P4HA1* axis, alleviating injury-induced miR-122-5p downregulation and consequent *P4HA1* upregulation. These results provide experimental evidence for a mechanistic basis of PTH's therapeutic application and highlight *P4ha1* as a promising biomarker for cirrhosis intervention.

See also the graphical abstract(Fig. 1).

## 1 Introduction

Liver cirrhosis is a serious liver disease that has caused over 1.32 million deaths, making it one of the leading causes of death globally (Huang et al., 2023[20]). The etiology of cirrhosis is complex and diverse, including chronic viral hepatitis, alcoholic liver disease, and non-alcoholic fatty liver disease (Acharya et al., 2017[1]). During disease progression, persistent liver injury can lead to liver failure, portal hypertension, and hepatocellular carcinoma, severely impacting patient survival (Sporea et al., 2013[38]). Current clinical management of cirrhosis is largely confined to etiological and supportive care, with limited pharmacological efficacy in advanced stages, often necessitating liver transplantation (Wang et al., 2023[44]). Therefore, in-depth investigation of the molecular pathogenesis of cirrhosis is not only crucial for developing safe and effective new drugs but also represents an urgent need to address current therapeutic gaps (Juanola et al., 2025[22]).

Traditional Chinese medicine has accumulated extensive clinical experience in the prevention and treatment of liver diseases, characterized by multi-target, multi-pathway, and holistic regulatory features, offering unique insights and resources for cirrhosis treatment (Zheng et al., 2025[54]). Pien Tze Huang (PTH), a traditional Chinese medicine formula originating from a Ming Dynasty imperial secret recipe with over five centuries of history, is a nationally protected class A traditional Chinese medicine variety, and its traditional preparation techniques have been inscribed on the National Intangible Cultural Heritage list. This formula is primarily composed of precious medicinal materials including panax notoginseng, calculus bovis, snake gallbladder, and musk (Chen et al., 2006[6]). Modern pharmacological studies have demonstrated that it contains multiple bioactive compounds and exhibits diverse biological activities including anti-inflammatory, antioxidant, immunomodulatory, and hepatoprotective effects (Chen et al., 2024[7]; Chen, 2021[11]). Preclinical animal experiments have confirmed that PTH attenuates carbon tetrachloride (CCl_4_)-induced liver injury and fibrosis, with mechanisms potentially involving inhibition of the NF-κB pathway and regulation of the TGF-β/Smad signaling pathway (Wang et al., 2022[45]; Zhao et al., 2017[53]).

Advances in high-throughput sequencing technology have substantially expanded the scope of transcriptome research, enabling simultaneous detection of messenger RNA (mRNA) and various non-coding RNA species, including microRNA (miRNA), long non-coding RNA (lncRNA), and circular RNA (circRNA) (Bernardo et al., 2020[3]; Luthra et al., 2017[31]). These non-coding RNAs participate in physiological and pathological processes through multiple mechanisms, such as epigenetic modification, transcriptional regulation, and post-transcriptional regulation (DeOcesano-Pereira et al., 2020[12]; Toden et al., 2021[40]). Among these, the competing endogenous RNA (ceRNA) regulatory mechanism has garnered considerable attention: mRNAs, lncRNAs, and circRNAs can compete with each other through shared miRNA response elements, forming complex regulatory networks that modulate target gene expression (Vitiello et al., 2020[43]). In the field of liver fibrosis, several studies have employed ceRNA networks to identify non-coding RNA molecules with potential diagnostic value (Zhang et al., 2024[51]). However, as the end-stage of liver fibrosis, the genome-wide ceRNA regulatory landscape of cirrhosis remains to be systematically characterized, and whether Pien Tze Huang exerts its anti-cirrhotic effects by modulating such networks is largely unexplored.

Based on the above background, the present study employed a CCl_4_-induced mouse model of cirrhosis, combined with whole transcriptome sequencing, to systematically investigate the molecular mechanisms underlying the anti-cirrhotic effects of PTH. Through integrated analysis incorporating differential expression analysis, functional enrichment analysis, ceRNA network construction, and multi-database cross-validation, we aimed to identify key targets and core regulatory axes responsible for the therapeutic effects of PTH against cirrhosis, thereby providing a theoretical basis for its clinical translation.

## 2 Materials and Methods

### Chemicals and reagents

Pien Tze Huang (PTH) powder was provided by Zhangzhou Pien Tze Huang Pharmaceutical Co., Ltd. (China National Medical Products Administration approval number: Z35020242). Carbon tetrachloride (CCl_4_, purity ≥ 99.5 %, Cat. No. C110623) and corn oil (Cat. No. C116025) were purchased from Shanghai Aladdin Biochemical Technology Co., Ltd. (Shanghai, China). TRIzol reagent (Cat. No. 15596026) was obtained from Thermo Fisher Scientific (USA). Hydrogen peroxide (H_2_O_2_, 3 % solution for microbiology; Cat. No. H755825) was purchased from Shanghai Aladdin Biochemical Technology Co., Ltd. Assay kits for alanine aminotransferase (ALT, Cat. No. C009-2-1), aspartate aminotransferase (AST, Cat. No. C010-2-1), and alkaline phosphatase (ALP, Cat. No. A059-2-2) were all purchased from Nanjing Jiancheng Bioengineering Institute (Nanjing, China).

### Drug preparation and quality control

Quality control for PTH powder was performed according to the standards of the Chinese Pharmacopoeia (2025 edition), and the presence of its main characteristic components (such as notoginsenosides and ginsenosides) was confirmed by reference to previous HPLC-MS analysis results (Zhu et al., 2021[55]). For animal experiments, the powder was dissolved in distilled water to prepare a 50 mg/mL stock solution for subsequent use. Carbon tetrachloride (CCl_4_) was mixed with corn oil at a ratio of 1:9 (v/v) to prepare a 10 % (v/v) CCl_4_ working solution. For cellular experiments, the powder was dissolved in sterile PBS to prepare a 30 mg/mL stock solution. The solution was heated in a 75 °C water bath for 30 min, followed by ultrasonication for 30 min. After centrifugation at 3000 rpm for 10 min, the supernatant was filtered sequentially through a 70 μm cell strainer and a 0.22 μm syringe filter to ensure sterility. The stock solution was aliquoted and stored at −20 °C until use.

### Animal experiments

Eighteen male C57BL/6 mice, aged 6 weeks and of SPF grade, were purchased from Shanghai Jiesijie Laboratory Animal Co., Ltd. (Shanghai, China). The animals were housed under controlled conditions at 21-22 °C with 50-55 % humidity and a 12/12 h light-dark cycle, with free access to food and water. All animal experimental procedures were approved by the Institutional Animal Care and Use Committee of Shanghai Jiao Tong University (Approval No. IACUC-2017-0033) and conducted in accordance with the ARRIVE guidelines.

The 18 mice were randomly divided into three groups (n = 6): Control group, Cirrhosis group, and Cirrhosis-PTH treatment group (Cirrhosis-PTH). Mice in the Cirrhosis and Cirrhosis-PTH groups received intraperitoneal injections of 10 % CCl_4_ (10 µL/g body weight) twice weekly for 12 consecutive weeks to induce the cirrhosis model; mice in the Control group received intraperitoneal injections of an equal volume of corn oil. Concurrently, mice in the Cirrhosis-PTH group received daily intragastric administration of PTH (0.25 mg/g body weight), while mice in the Control and Cirrhosis groups received an equal volume of distilled water by gavage. All treatments were continued for 12 weeks. After the final administration, mice were anesthetized with isoflurane and euthanized by cervical dislocation. Blood samples were collected from the orbital sinus to obtain serum, and liver tissue samples were either preserved in 10 % formalin or stored at -80 °C for subsequent analysis.

### Serum biochemical parameter detection

Serum levels of ALT, AST, and ALP were measured using an automated biochemical analyzer. The liver index (liver weight/body weight × 100 %) was calculated.

### Histological analysis

Liver tissues were fixed in 4 % paraformaldehyde, embedded in paraffin, and sectioned into 4 μm slices. Hematoxylin-eosin (HE) staining, Masson's trichrome staining, and Sirius Red staining were performed respectively. Stained sections were observed and photographed under a light microscope.

### RNA extraction and sequencing

Total RNA was extracted from liver tissues using TRIzol reagent. RNA purity was assessed by A260/A280 ratio, with all samples showing ratios > 1.9. After ribosomal RNA depletion, two types of sequencing libraries were constructed: lncRNA, circRNA, and mRNA sequencing libraries were prepared using the NEBNext RNA Library Prep Kit, while miRNA sequencing libraries were prepared using the VAHTS Small RNA Library Prep Kit. The lncRNA/circRNA/mRNA libraries were sequenced on the Illumina HiSeq 4000 platform with 150 bp paired-end reads, and the miRNA libraries were sequenced on the Illumina NovaSeq platform with 50 bp paired-end reads.

### Sequencing data quality control

FastQC (Brown et al., 2017[4]) (v0.11.9) was used to assess raw sequencing quality. For mRNA and lncRNA data, adapter sequences, poly-N sequences, and low-quality reads were removed using in-house Perl scripts to obtain clean reads. Clean reads were aligned to the mouse reference genome (GRCm38) using hisat2 (Kim et al., 2019[24]) (v2.2.1), and transcript assembly and quantification were performed using stringtie (Pertea et al., 2015[33]) (v2.1.5) to obtain the FPKM expression matrix. For miRNA data, Cutadapt (Kechin et al., 2017[23]) (v4.2) was used to remove adapters and sequences shorter than 10 nt, and the processed sequences were aligned to the mouse reference genome (GRCm38) using Bowtie (Langmead and Salzberg, 2012[27]) (v1.3.1) and miRDeep2 (Friedländer et al., 2012[16]) for miRNA quantification. For circRNA data, Trimgalore (Sun, 2020[39]) and Fastp (Chen et al., 2018[8]) were used to remove adapters, poly-N sequences, and low-quality reads, Bowtie2 (Langdon, 2015[26]) was used to remove rRNA and E. coli contamination sequences, and CIRI2 (Li et al., 2021[29]) was used for circRNA identification and quantification. We confirm that all RNA-seq libraries were prepared in a single experiment using the same batch of reagents, pooled, and then loaded across lanes of the same flow cell for sequencing. No batch effect was observed, and therefore batch effect correction was not required.

### Differential expression analysis

Differential expression analysis of mRNA, lncRNA, miRNA, and circRNA was performed using the DESeq2 (Varet et al., 2016[42]) (v1.40.1) R package. Significance thresholds were set as follows: for mRNA and lncRNA, |log_2_FC| > 1 and adjusted *P*-value < 0.01; for miRNA, |log_2_FC| > 1 and *P*-value < 0.05; for circRNA, |log_2_FC| > 0.25 and *P*-value < 0.05. Data visualization and heatmap generation were performed using the ggplot2 (Cao et al., 2023[5]) (v3.4.2) and pheatmap (Gaujoux and Seoighe, 2010[17]) R packages. Principal component analysis (PCA) and hierarchical clustering analysis were performed based on the mRNA expression profiles of all samples.

### Functional enrichment analysis

Gene Ontology (GO) and Kyoto Encyclopedia of Genes and Genomes (KEGG) pathway enrichment analyses of differentially expressed mRNAs were performed using the clusterProfiler (Wu et al., 2021[49]) (v4.4.4) R package. The significance threshold for enrichment was set at *P*-value < 0.05.

### Drug similarity analysis

Drug similarity analysis was performed based on the Connectivity Map (CMap) database. Mouse gene symbols of differentially expressed genes in the Cirrhosis-PTH vs. Cirrhosis comparison were converted to human homologous gene symbols and divided into up-regulated and down-regulated gene sets. Due to the input limitations of the CLUE query tool (Lamb, 2007[25]), all up-regulated genes and the top 150 down-regulated genes ranked by |log_2_FC| value were selected for query. The PTH-induced gene expression profile was compared with drug-induced gene expression profiles in the CMap database, and connectivity scores (ranging from-100 to 100) were calculated, with higher scores indicating stronger similarity. The analysis results from human liver-derived cell lines (HEPG2, PHH, and HUH7) were specifically focused on.

### Construction of ceRNA networks

To investigate whether non-coding RNAs participate in the regulatory effects of PTH on cirrhosis through the ceRNA mechanism, we integrated database prediction and differential expression analysis strategies. First, Miranda, Targetscan, and RNAhybrid (Enright et al., 2003[15]; Shi et al., 2017[37]; Zhang et al., 2018[52]) were used to predict potential target miRNAs of DEcircRNAs, as well as potential target mRNAs of DEmiRNAs and DElncRNAs. However, no target miRNAs associated with DElncRNAs were identified in this study. Subsequently, the predicted interaction pairs were integrated with the differential expression analysis results, retaining only those pairs in which all molecules were differentially expressed in the corresponding comparison groups. Based on this, condition-specific ceRNA networks were constructed for the Cirrhosis vs. Control and Cirrhosis-PTH vs. Cirrhosis comparisons, respectively.

### Human proteome data analysis

We used the protein abundance and gene expression data from 32 human tissues published in the study by Jiang et al. (Jiang et al., 2020[21]) to query the protein abundance and tissue-specific expression of candidate biomarker genes in different human tissues. The Therapeutic Target Database (TTD, https://db.idrblab.net/ttd/) was then searched to identify whether candidate biomarker genes associated with cirrhosis have been previously reported (Chen et al., 2002[9]).

### Cell culture and treatment

Human HepG2 cells, obtained from the Cell Bank of the Chinese Academy of Sciences (Shanghai, China), were cultured in Dulbecco's Modified Eagle Medium (DMEM, Gibco, USA) supplemented with 10 % fetal bovine serum (FBS, Gibco, USA), 100 U/mL penicillin and 100 µg/mL streptomycin (Gibco, USA) at 37 °C in a 5 % CO_2_ atmosphere. The culture medium was routinely replaced, and cells were either subcultured or used in experiments when they reached 80-90 % confluence. Cells were treated with different concentrations of PTH (0.05, 0.1, and 0.2 mg/mL) for 24 h based on a previous study (Liu et al., 2026[30]).

### Cell viability assay

HepG2 cells (2 × 10^3^ cells/mL) were seeded in 96-well plates and treated with PTH (0.05, 0.1, and 0.2 mg/mL) for 24 h, followed by exposure to a gradient of H_2_O_2_ concentrations (200-1000 μM) for 2 h. Control cells were treated with vehicle alone or PTH alone without H_2_O_2_ exposure. Cell viability was assessed by incubating the cells with 10 μL of CCK-8 solution (Beyotime, Shanghai, China) for 1 h following the manufacturer's protocol, and the absorbance was measured at 450 nm using a microplate reader.

### Inhibition of miR-122-5p by miRNA inhibitor transfection

The hsa-miR-122-5p inhibitor was purchased from MedChemExpress (Shanghai, China). HepG2 cells were seeded in 6-well plates and transfected with 100 nM of the inhibitor using Lipofectamine 3000 (Thermo Fisher Scientific) according to the manufacturer's instructions. Approximately 6 h after transfection, the medium was replaced with fresh complete medium containing serum and antibiotics. The cells were then incubated for an additional 48 h before subsequent analysis. The transfected cells were divided into the following four groups: a control group without any treatment, a negative control inhibitor group (NC inhibitor), an miR-122-5p inhibitor group, and an miR-122-5p inhibitor plus PTH group.

### Real-time quantitative PCR (RT-qPCR)

Total RNA (including miRNA) was extracted from HepG2 cells using the SPARKeasy Improved Tissue/Cell RNA Kit (SparkJade, Shanghai, China; Cat. No. AC0202). For mRNA detection, reverse transcription was performed using the BeyoRT™ III First-Strand cDNA Synthesis Mix (Beyotime, Shanghai, China; Cat. No. D7182). For miRNA detection, reverse transcription was carried out using the miRNA 1st Strand cDNA Synthesis Kit (by stem-loop) (Vazyme, Nanjing, China; Cat. No. MR101-02). qPCR was performed using the BIO-RAD CFX96 Real-Time PCR Detection System with TruQuant SYBR Green (Thermo Fisher Scientific, USA; Cat. No. FB05001010). The primer sequences are listed in Supplementary Table 1. Gene expression levels were normalized to GAPDH (for mRNA) and U6 (for miRNA), and relative expression was calculated using the 2^-^ΔΔCt method.

### Statistical analysis

All data are presented as mean ± standard error of the mean (SEM). Comparisons among multiple groups were performed using one-way analysis of variance (ANOVA) followed by Tukey's post hoc test. A *P*-value < 0.05 was considered statistically significant. Statistical analyses were performed using R software (v4.1.0).

## 3 Results

### PTH treatment attenuates CCl_4_-induced liver injury and cirrhosis in mice

**Figure 2:**
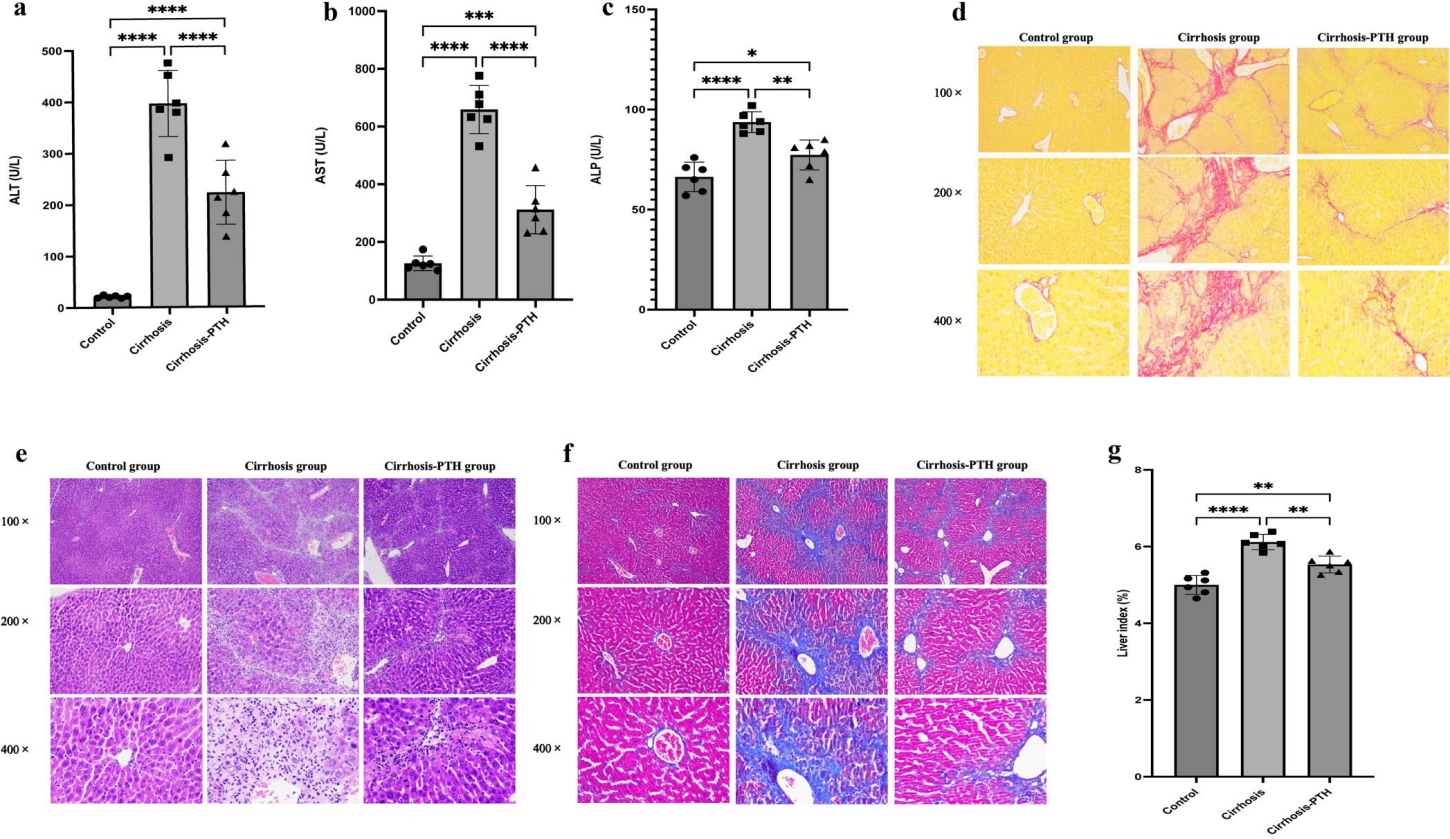
Effects of PTH on CCl_4_-induced liver injury and cirrhosis in mice: (A-C) Serum levels of ALT, AST, and ALP in each group (n = 6 per group); (D-F) Representative histological staining of liver sections from each group: Sirius Red staining (D), HE staining (E), and Masson staining (F); scale bar, 100 μm; (G) Liver index (liver weight/body weight × 100 %) for each group. Data are presented as the mean ± SEM. *P < 0.05, **P < 0.01, ***P < 0.001, ****P < 0.0001

Compared with the Control group, mice in the Cirrhosis group exhibited significantly elevated serum levels of ALT and AST, indicating marked hepatocellular damage and inflammatory response. PTH treatment significantly reduced both ALT and AST levels (Figure 2A, B(Fig. 2)). Furthermore, serum levels of ALP were markedly increased in cirrhotic mice and were significantly reduced following PTH treatment (Figure 2C(Fig. 2)). Histological analysis further supported these conclusions. HE staining demonstrated that PTH treatment significantly attenuated the severe cirrhotic morphology and cellular disorganization observed in cirrhotic mice. Masson staining and Sirius Red staining revealed extensive collagen fiber deposition and pseudolobule formation in the livers of cirrhotic mice, both of which were significantly reduced following PTH treatment (Figure 2D-F(Fig. 2)). Finally, compared with Control mice, the liver index was significantly decreased in the Cirrhosis group, reflecting liver atrophy and functional impairment. PTH intervention significantly improved this, indicating partial recovery of liver mass (Figure 2G(Fig. 2)). Taken together, these results show that PTH treatment ameliorates CCl_4_-induced liver injury and cirrhosis in mice.

### PTH reverses global transcriptomic alterations in cirrhotic liver

**Figure 3:**
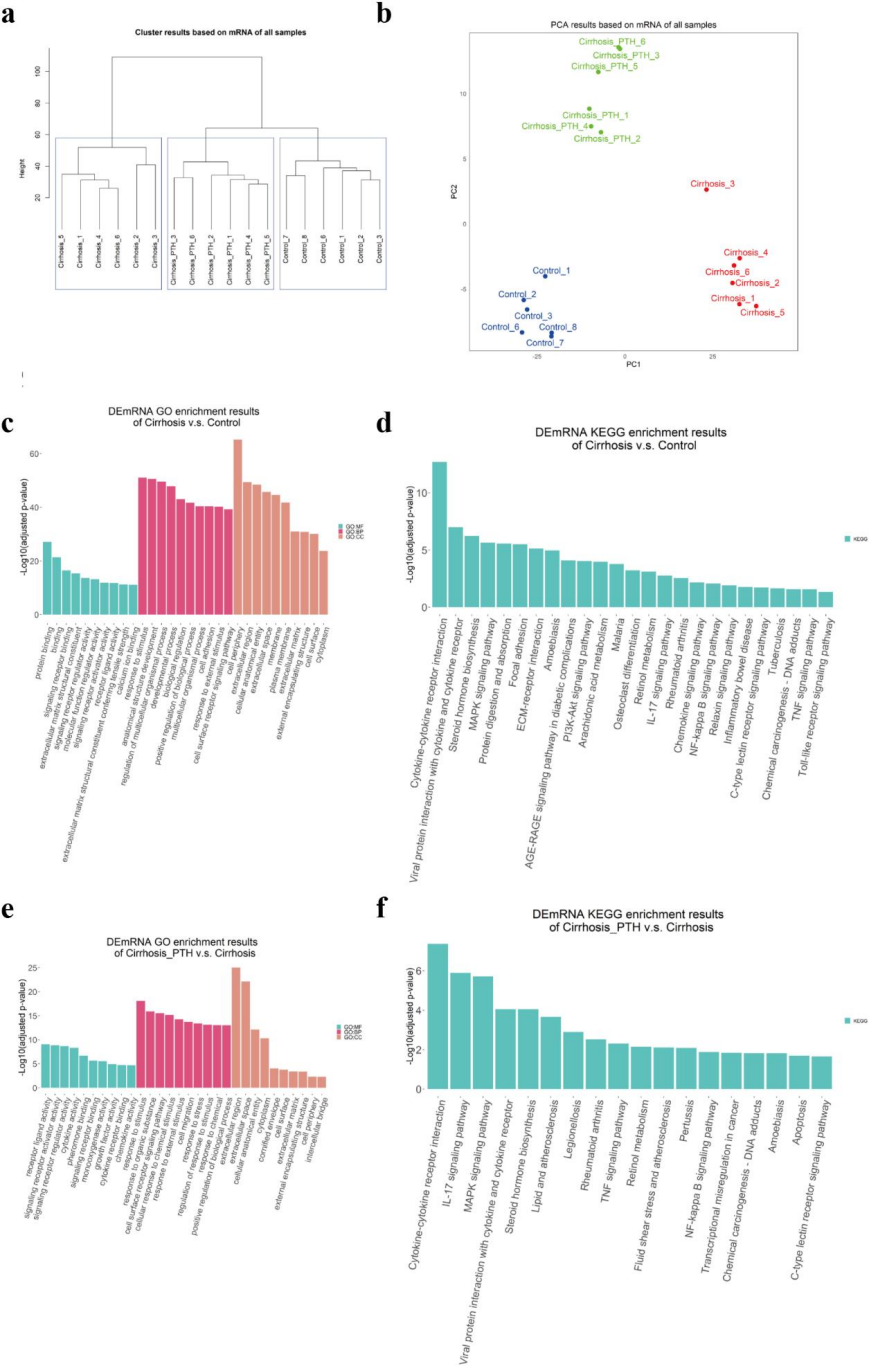
Sample cluster result based on gene expression of mRNAs and gene functional analysis of differentially expressed mRNAs: (A) Cluster results based on mRNAs of all samples; (B) PCA results based on mRNAs of all samples; (C) Top GO enrichment results of DEGs of Cirrhosis vs. Control; (D) Top KEGG enrichment results of DEGs of Cirrhosis vs. Control; (E) Top GO enrichment results of DEGs of Cirrhosis- PTH vs. Cirrhosis; (F) Top KEGG enrichment results of DEGs of Cirrhosis-PTH vs. Cirrhosis

Differential expression analysis revealed that in the Cirrhosis vs. Control comparison, 3,231 mRNAs were significantly differentially expressed. In the Cirrhosis-PTH vs. Cirrhosis comparison, 1,689 mRNAs were identified as differentially expressed (Supplementary Figure 1). PCA of mRNA expression profiles showed clear separation of the three groups, with the Cirrhosis-PTH group clustering closer to Control than to Cirrhosis (Figure 3A(Fig. 3)). Hierarchical clustering analysis confirmed this pattern, demonstrating that the gene expression signature of PTH-treated mice was more similar to controls (Figure 3B(Fig. 3)). To identify genes specifically reversed by PTH treatment, we intersected the differentially expressed genes (DEGs) from the two comparisons. A total of 1,518 genes were dysregulated in cirrhosis and significantly reversed by PTH treatment. The log_2_ fold changes of these overlapping genes showed a strong negative correlation between the two comparisons (r = -0.925, *P *< 2.2e-16), indicating that PTH treatment was associated with transcriptional changes opposite to those observed in the disease condition.

### PTH modulates inflammatory and fibrotic pathways in cirrhotic liver

To characterize the biological processes associated with cirrhosis and its treatment by PTH, we performed Gene Ontology (GO) and Kyoto Encyclopedia of Genes and Genomes (KEGG) pathway enrichment analysis on DEGs from both comparisons (see Figure 3C-F(Fig. 3)). In the Cirrhosis vs. Control comparison, GO analysis revealed enrichment of terms related to "protein binding" (GO:0005515), "response to stimulus" (GO:0050896), and "cell periphery" (GO:0071944). KEGG pathway analysis identified 25 significantly enriched pathways (*P* < 0.05), including Cytokine-cytokine receptor interaction (mmu04060), MAPK signaling pathway (mmu04010), NF-κB signaling pathway (mmu04064), and PI3K-Akt signaling pathway (mmu04151). In the Cirrhosis-PTH vs. Cirrhosis comparison, GO analysis showed enrichment of terms including "receptor ligand activity" (GO:0048018), "response to stimulus" (GO:0050896), and "extracellular region" (GO:0005576). KEGG analysis revealed significant enrichment of pathways including Cytokine-cytokine receptor interaction (mmu04060), IL-17 signaling pathway (mmu04657), MAPK signaling pathway (mmu04010), TNF signaling pathway (mmu04668), and NF-κB signaling pathway (mmu04064).

To further explore the clinical relevance of these pathway-level changes, we performed CMap analysis using the 1,518 PTH-responsive genes. The PTH-induced transcriptomic signature showed significant similarity to that of statins (simvastatin and atorvastatin) and beta-blockers (metoprolol and propranolol), all of which are drugs currently in clinical trials for cirrhosis (Supplementary Tables 2-4). This convergence between pathway enrichment and drug similarity predictions provides additional support for the potential therapeutic relevance of PTH in cirrhosis.

### Construction of ceRNA networks

To explore potential regulatory interactions among differentially expressed coding and non-coding RNAs, we first analyzed the expression profiles of non-coding RNAs. In the Cirrhosis vs. Control comparison, 491 lncRNAs, 137 miRNAs, and 214 circRNAs were significantly differentially expressed (Supplementary Figure 2). In the Cirrhosis-PTH vs. Cirrhosis comparison, 218 lncRNAs, 61 miRNAs, and 145 circRNAs were identified as differentially expressed (Supplementary Figure 3). These differentially expressed non-coding RNAs, together with the previously identified DEmRNAs, provided the basis for subsequent ceRNA network construction.

We constructed condition-specific ceRNA networks by integrating DEcircRNAs, DEmiRNAs, and DEmRNAs. In the Cirrhosis vs. Control comparison, the ceRNA network comprised 130 DEcircRNAs, 29 DEmiRNAs, and 31 DEmRNAs (Supplementary Figure 4A, Supplementary Table 5). In the Cirrhosis-PTH vs. Cirrhosis comparison, the network comprised 52 DEcircRNAs, 10 DEmiRNAs, and 16 DEmRNAs (Supplementary Figure 4B, Supplementary Table 6).

### PTH regulates a core ceRNA network centered on the miR-122-5p-P4ha1 axis

**Figure 4:**
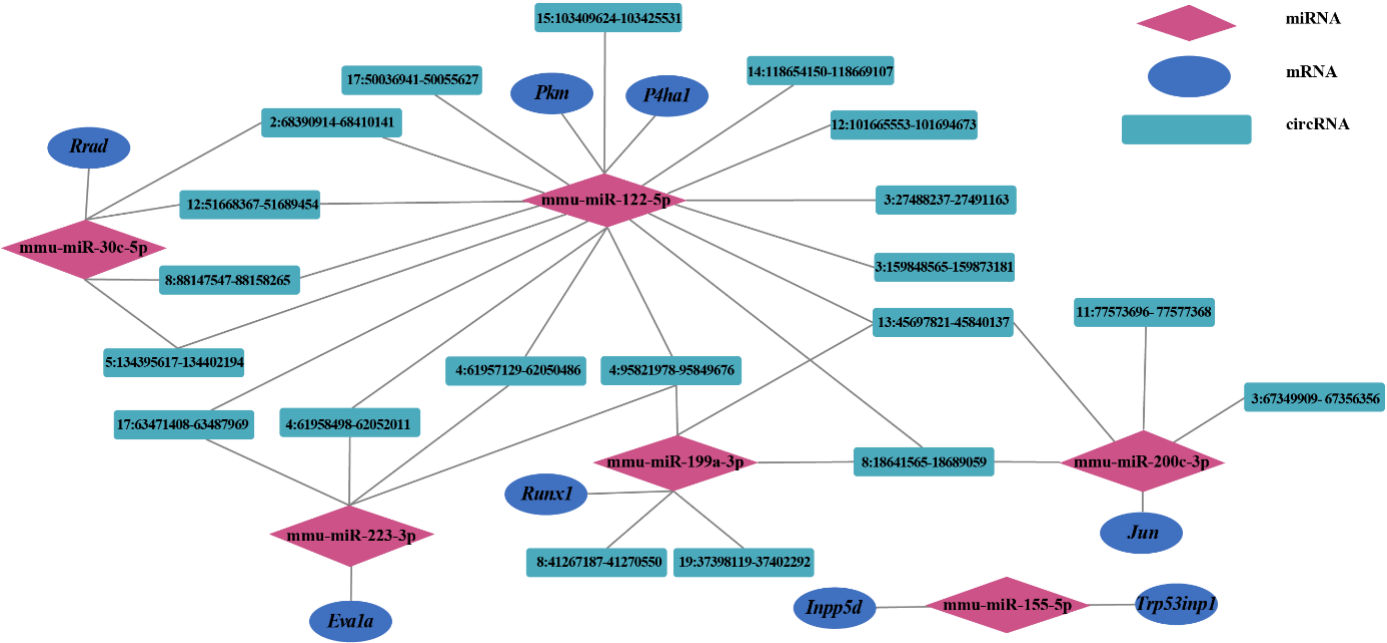
Core ceRNA network identified by intersecting condition-specific networks

**Figure 5:**
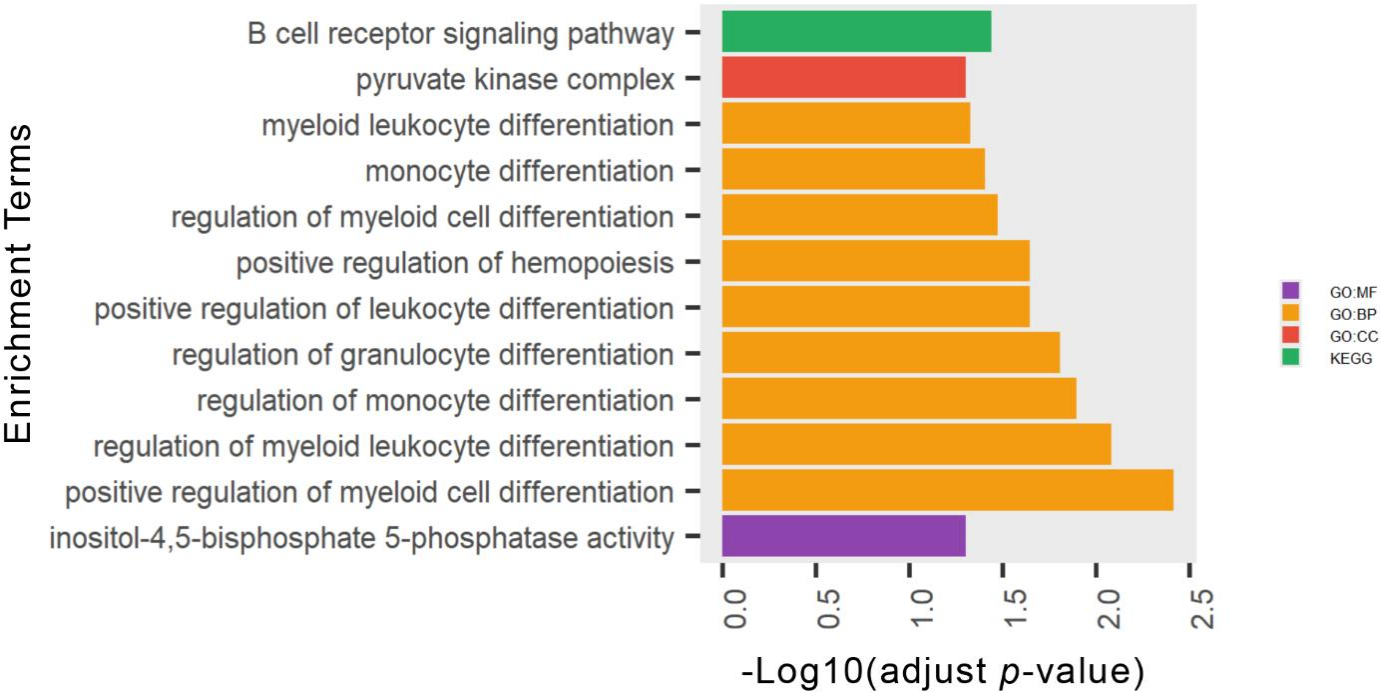
GO and KEGG pathway enrichment analysis results of eight selected DEmRNAs

To identify key regulatory molecules underlying the anti-cirrhotic effect of PTH, we intersected the two condition-specific ceRNA networks and applied a stringent filter requiring reversed expression trends, specifically molecules that were dysregulated in cirrhosis and normalized by PTH treatment. This analysis identified a core regulatory module comprising 6 DEmiRNAs, 8 DEmRNAs, and 27 DEcircRNAs. The six core miRNAs were mmu-miR-122-5p, mmu-miR-155-5p, mmu-miR-199a-3p, mmu-miR-200c-3p, mmu-miR-223-3p, and mmu-miR-30c-5p (Figure 4(Fig. 4)). Among these, mmu-miR-122-5p exhibited the highest connectivity within the network, targeting two mRNAs (*P4ha1* and *Pkm*) and interacting with 13 circRNAs. Notably, mmu-miR-122-5p is the most abundant and liver-specific miRNA (Bandiera et al., 2015[2]). The eight core mRNAs were *P4ha1, Runx1, Inpp5d, Trp53inp1, Rrad, Pkm, Eva1a*, and *Jun*. Functional enrichment analysis of these eight genes revealed significant enrichment of inositol-4,5-bisphosphate 5-phosphatase activity (GO:0030487), pyruvate kinase complex (GO:1902912), and the B cell receptor signaling pathway (KEGG: map04662). These genes were also associated with the activation of innate immune cells, including leukocytes and granulocytes, suggesting their involvement in immune regulation (Figure 5(Fig. 5)).

### Multi-omics and clinical evidence support P4HA1 as a therapeutic target in cirrhosis

**Figure 6:**
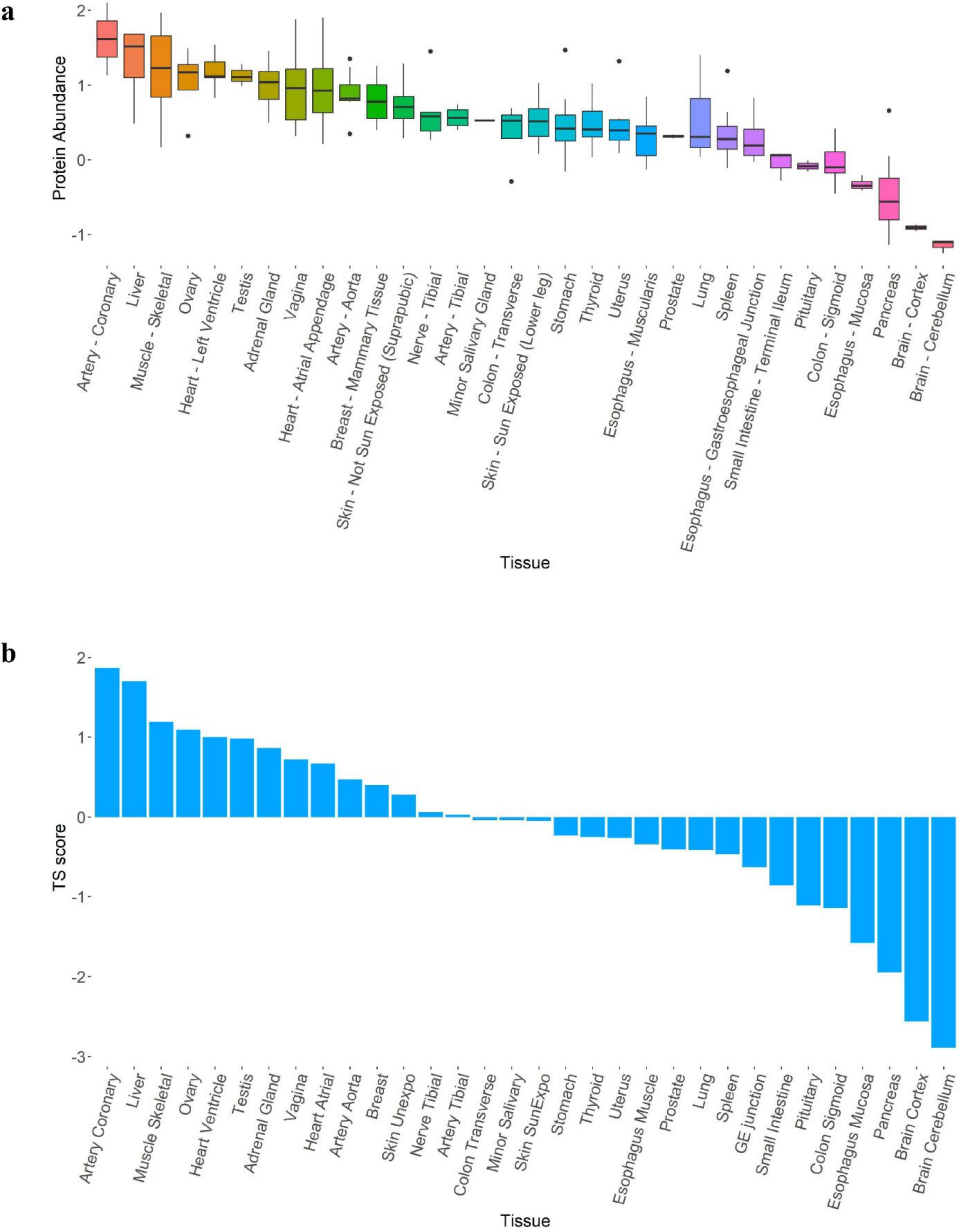
Protein abundance and TS score of *P4HA1* in different tissues from the TTD database: (A) Protein abundance of *P4HA1* in different tissues; (B) TS score of *P4HA1* in different tissues

To assess the translational relevance of these findings, we investigated the distribution of human homologous genes of the above predicted DEmRNAs (*P4ha1, Runx1, Inpp5d, Trp53inp1, Rrad, Pkm, Eva1a, *and *Jun*) in human tissues based on the dataset provided by Jiang et al. We found that *P4HA1*, the human homolog of *P4ha1*, ranked second in protein abundance in the liver among all 32 tissues examined. Furthermore, the tissue specificity score of *P4HA1* also ranked second among all 32 tissues (Figure 6(Fig. 6)). Except for *P4HA1*, the protein abundance of six genes (*RUNX1, INPP5D, RRAD, PKM, EVA1A*, and *JUN*) was at low levels in the liver (see Supplementary Figure 5). Consistent with this liver-enriched expression pattern, *P4HA1* is a known target of Lufironil (also known as HOE 077), a small molecule inhibitor of prolyl 4-hydroxylase that has entered Phase II clinical trials for cirrhosis (TTD, Target ID: T61729; Drug ID: D01NCH).

### PTH regulates the miR-122-5p/P4HA1 axis in vitro

**Figure 7:**
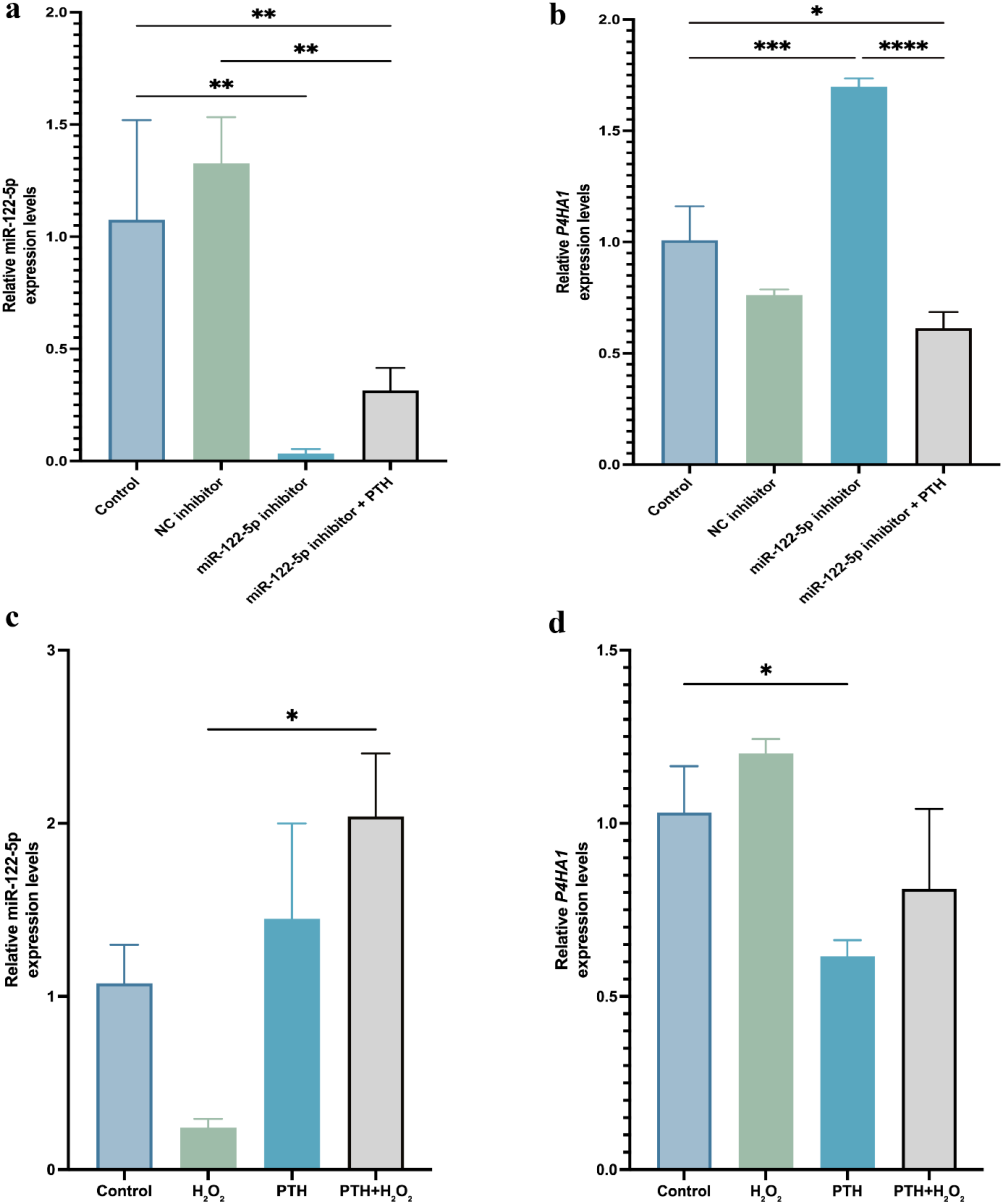
PTH modulates the miR-122-5p/*P4HA1* axis in HepG2 cells: (A-B) HepG2 cells were transfected with miR-122-5p inhibitor (100 nM) or negative control (NC inhibitor), followed by treatment with or without PTH (0.1 mg/mL) as indicated; (A) miR-122-5p expression levels measured by qPCR; (B) *P4HA1* mRNA expression levels measured by qPCR; (C-D) HepG2 cells were pretreated with or without PTH (0.1 mg/mL) for 24 h, followed by exposure to H_2_O_2_ (400 μM) or vehicle control for 2 h; (C) miR-122-5p expression levels measured by qPCR; (D) *P4HA1* mRNA expression levels measured by qPCR. Data are presented as mean ± SEM (n = 3 independent experiments). *P < 0.05, **P < 0.01, ***P < 0.001, ****P < 0.0001

Based on dose-response experiments, 0.1 mg/mL PTH and 400 μM H_2_O_2_ were selected for subsequent validation (Supplementary Figure 6). To directly test whether miR-122-5p regulates *P4HA1*, HepG2 cells were transfected with 100 nM miR-122-5p inhibitor or negative control. Transfection with miR-122-5p inhibitor significantly decreased miR-122-5p expression, confirming successful knockdown (Figure 7A(Fig. 7)). *P4HA1* mRNA expression was significantly increased in the inhibitor group and was significantly decreased following PTH co-treatment (Figure 7B(Fig. 7)). In an oxidative stress-induced liver injury model, HepG2 cells were treated with H_2_O_2_. H_2_O_2_ treatment decreased miR-122-5p expression and increased* P4HA1* expression (Figure 7C-D(Fig. 7)). PTH pretreatment significantly increased miR-122-5p expression compared to the H_2_O_2_ group (Figure 7C(Fig. 7)).

See also the supplementary data.

## 4 Discussion

Liver cirrhosis is the advanced stage of chronic liver diseases, characterized by the formation of pseudolobules and disruption of normal liver architecture (Acharya et al., 2017[1]). While liver fibrosis has been extensively studied, the molecular mechanisms underlying established cirrhosis remain poorly understood. In the present study, we employed a CCl_4_-induced mouse model of cirrhosis to evaluate the therapeutic potential of Pien Tze Huang (PTH) and to investigate its molecular mechanisms through whole transcriptome sequencing and integrative bioinformatics analysis. Of note, the selected PTH dose of 250 mg/kg was based on clinical dose conversion (182-273 mg/kg mouse equivalent) and an independent dose optimization study (234 mg/kg) (Gou et al., 2023[18]; Liu et al., 2026[30]), and was previously validated to have no observable toxicity (Di et al., 2022[13]).

The pathological features of cirrhosis include hepatocellular injury, cholestasis, and collagen deposition. The CCl_4_ regimen employed in this study (1.0 mL/kg of a 10 % solution, 10 µL/g, twice weekly for 12 weeks) represents a moderate-dose protocol widely used for cirrhosis induction. Histological analysis confirmed typical pseudolobule formation and significant collagen deposition, indicating successful establishment of cirrhosis. No mortality was observed throughout the experiment. Under these experimental conditions, serum biochemical parameters showed that PTH treatment significantly reduced the elevated levels of ALT, AST, and ALP in cirrhotic mice, and partially restored the liver index, indicating that PTH alleviates hepatocellular injury and cholestasis. Macroscopic observation and histological analysis further confirmed that PTH treatment improved liver morphology and reduced collagen fiber deposition and pseudolobule formation. These findings are consistent with previously reported hepatoprotective effects of PTH (Wang et al., 2022[45]; Zhao et al., 2017[53]), providing a reliable phenotypic basis for subsequent transcriptomic mechanistic studies.

Transcriptomic sequencing analysis revealed that PTH treatment induced significant changes in the gene expression profile of cirrhotic livers. Both PCA and hierarchical clustering analyses indicated a tendency for the transcriptomic profile of the PTH-treated group to shift toward that of the Control group, suggesting that PTH may have the potential to partially counteract disease-associated transcriptional alterations. Differential expression analysis identified 1,518 genes that were dysregulated in cirrhosis and showed significant changes upon PTH treatment. The log_2_ fold changes of these overlapping genes exhibited a notable negative correlation between the two comparisons, further supporting the potential remodeling effect of PTH at the transcriptomic level.

To understand the biological significance underlying these transcriptional changes, we performed functional enrichment analysis on the differentially expressed genes from both comparisons. In the Cirrhosis vs. Control comparison, enriched pathways included cytokine-cytokine receptor interaction, MAPK signaling pathway, NF-κB signaling pathway, and PI3K-Akt signaling pathway, all of which are closely associated with liver fibrosis, hepatocyte apoptosis, and inflammatory responses (Du et al., 2023[14]; Melis et al., 2024[32]; Sadowska et al., 2023[35]), consistent with the known molecular mechanisms of cirrhosis. This finding validates the biological relevance of the CCl_4_-induced model at the transcriptomic level. In the Cirrhosis-PTH vs. Cirrhosis comparison, enrichment analysis revealed that PTH treatment similarly enriched cytokine-cytokine receptor interaction, MAPK signaling pathway, and NF-κB signaling pathway, suggesting that PTH may exert its therapeutic effects by modulating these pathways critically involved in cirrhosis pathogenesis. Furthermore, the IL-17 signaling pathway and TNF signaling pathway were also significantly enriched among PTH-responsive genes. These results indicate that the anti-cirrhotic effects of PTH may involve multi-faceted regulation of inflammatory signaling pathways. To further deepen our understanding at the pathway level, we performed CMap analysis using the 1,518 PTH-responsive genes. The results showed that the PTH-induced transcriptomic signature exhibited certain similarities to that of statins (simvastatin and atorvastatin), which are currently in clinical trials for cirrhosis, and beta-blockers (metoprolol and propranolol), which are already widely used in the clinical management of cirrhosis (Supplementary Tables 2-4). Notably, these drugs exert their effects through modulation of inflammation and fibrosis-related pathways (Turco et al., 2023[41]; Wu and Lin, 2024[47]), which parallels the enrichment of inflammatory pathways such as IL-17, TNF, and NF-κB observed among PTH-responsive genes. This suggests a potential mechanistic similarity, providing a preliminary bioinformatics basis for further investigation of PTH's therapeutic potential.

Gene expression is subject to dual regulation at both transcriptional and post-transcriptional levels. In liver fibrosis research, liver-enriched miRNAs such as miR-122 and the miR-29 family have been demonstrated to regulate fibrotic processes by targeting collagen-related genes (Hu et al., 2012[19]; Roderburg et al., 2011[34]). We first analyzed the expression profiles of non-coding RNAs and found that numerous miRNAs and circRNAs were dysregulated under cirrhotic conditions, with partial restoration of their expression following PTH treatment. Based on these differentially expressed non-coding RNAs, we constructed condition-specific ceRNA networks under disease and treatment states.

By comparing the two networks and screening for molecules with reversed expression trends, we identified a core regulatory module consisting of 6 miRNAs, 8 mRNAs, and 27 circRNAs. This module represents a core regulatory axis that is dysregulated in cirrhosis and reversed by PTH treatment, serving as a candidate target through which PTH may exert its effects via the ceRNA mechanism. Notably, mmu-miR-122-5p exhibited the highest connectivity within the network, targeting two mRNAs (*P4ha1* and *Pkm*) and interacting with 13 circRNAs. miR-122-5p is the most abundant and liver-specific miRNA and has been widely reported to participate in the regulation of liver fibrosis, lipid metabolism, and hepatocellular carcinoma (Wen and Friedman, 2012[46]; Zeng et al., 2015[50]). A previous study demonstrated that miR-122 directly targets *P4HA1* and negatively regulates collagen production in hepatic stellate cells (Li et al., 2013[28]), suggesting a potential regulatory link between miR-122 and *P4HA1* in fibrotic conditions. Our findings raise the possibility that this regulatory axis may also be relevant in the cirrhotic stage, as both miR-122 and *P4ha1* were identified as key nodes in the PTH-responsive ceRNA network. Functional enrichment analysis of the eight core mRNAs revealed that these genes are involved in immune regulation and metabolic processes. This finding corroborates the inflammatory pathway regulation observed in the pathway enrichment analysis, suggesting that PTH may exert multi-target regulatory effects through the ceRNA network.

Among the eight core mRNAs, *P4ha1* emerged as a candidate of particular interest due to its unique biological function. *P4ha1* encodes the alpha subunit of prolyl 4-hydroxylase, a rate-limiting enzyme in collagen biosynthesis that catalyzes the hydroxylation of proline residues in collagen chains, thereby ensuring proper triple helix formation and secretion (Chen et al., 2006[6]). Excessive collagen deposition is a pathological hallmark of cirrhosis, mechanistically linking *P4ha1* to this disease.* P4ha1 *occupies a distinctive position within the ceRNA network, being targeted by the liver-specific miR-122-5p-the miRNA with the highest connectivity in the network, interacting with 13 circRNAs. This regulatory axis suggests that *P4ha1* expression may be subject to liver-restricted post-transcriptional regulation. Human proteome data revealed that *P4HA1* ranks second in protein abundance in the liver among all 32 tissues examined, with an equally high tissue specificity score. In contrast, the other six core genes exhibited low expression levels in the liver, suggesting they may primarily function in extrahepatic tissues or other physiological processes. From a drug target perspective, *P4HA1* has been validated as a therapeutic target in anti-fibrotic strategies. This is exemplified by Lufironil (HOE 077), a small-molecule inhibitor that directly suppresses* P4ha1* enzymatic activity and has previously advanced to Phase II clinical trials for cirrhosis (TTD: T61729/D01NCH) (Sakaida et al., 1996[36]).

Our experimental validation confirms the functional relevance of the *P4HA1*-centered ceRNA regulatory network in liver cells. Transfection with miR-122-5p inhibitor significantly reduced miR-122-5p expression and concurrently increased *P4HA1* mRNA levels, while PTH co-treatment attenuated this *P4HA1* upregulation. This inverse relationship is consistent with previous reports identifying *P4HA1* as a direct target of miR-122-5p in other cell types (Wu et al., 2023[48]), supporting the presence of this regulatory axis in hepatocytes. Under oxidative stress conditions, which are known to promote hepatic stellate cell activation and cirrhosis progression, we observed that H_2_O_2_ treatment decreased miR-122-5p expression and increased *P4HA1 *levels. Notably, PTH pretreatment significantly attenuated the injury-induced downregulation of miR-122-5p and reduced the injury-associated increase in *P4HA1*. Interestingly, PTH treatment alone also significantly decreased *P4HA1* expression compared to the control group, suggesting that PTH may exert its protective effects in liver cirrhosis through modulating the *P4HA1*-centered ceRNA network. However, the precise molecular mechanisms by which PTH regulates the components of this ceRNA network remain to be fully elucidated. Further studies are needed to determine the upstream signaling pathways and the roles of circRNA-miRNA interactions in this process.

Our findings should be interpreted within the context of several limitations. First, the modest sample size (n = 6 per group) may limit the statistical power of our analyses, which is common in exploratory transcriptomic studies. Larger cohorts will be necessary to validate these findings. Second, while we applied stringent filtering to prioritize biologically coherent expression patterns, ceRNA network predictions still carry an inherent risk of false positives. Therefore, these interactions should be viewed as hypotheses to guide future functional studies. Third, while our *in vitro* experiments using HepG2 cells and H_2_O_2_-induced oxidative stress offer preliminary support for the miR-122-5p/ *P4HA1* axis, this model does not fully recapitulate the complex microenvironment of liver cirrhosis. Future studies employing more advanced models (e.g., *in vivo* models) are needed to further elucidate this axis in cirrhosis pathogenesis.

In conclusion, this study employed a progressive integrative strategy to systematically elucidate the mechanisms by which PTH reverses transcriptomic abnormalities in cirrhosis. Whole transcriptome sequencing confirmed the regulatory effects of PTH on key inflammatory and fibrotic pathways, and ceRNA network analysis identified the miR-122-5p/*P4ha1* axis as a core effector pathway. Functional validation in hepatocytes further confirmed that PTH modulates this axis by alleviating injury-induced miR-122-5p downregulation and subsequent *P4HA1 *upregulation. To our knowledge, this represents the first study to establish a direct link between PTH and the miR-122-5p/*P4ha1* ceRNA axis in cirrhosis, providing new insights into the molecular mechanisms underlying PTH's protective effects. The identification of *P4ha1* provides a theoretical basis for its potential as a therapeutic target, suggesting that targeted intervention strategies combined with the modern development of PTH may offer new therapeutic options for patients with cirrhosis.

## Notes

Yunxiao Lin, Fan Yang, Yingtian Zhang, and Xiaoqin Zhang contributed equally as first author.

Zexiu Zhang and Shengying Qin (Bio-X Institutes, Key Laboratory for the Genetics of Developmental and Neuropsychiatric Disorders (Ministry of Education), Shanghai Jiao Tong University, 1954 Hua Shan Road, Shanghai 200030, P.R. China; E-mail: chinsir@sjtu.edu.cn; Tel.: 021-62932779) contributed equally as corresponding author.

## Declaration

### Acknowledgments

This research was supported by the Fundamental Research Funds for the Central Universities (YG2023LC13, YG2023QNA07), Natural Science Foundation of Fujian Province (2022J01530), National Nature Science Foundation of China (82003856, 82274025, 81773818, 81273596, 81503051), Sichuan Province science and technology plan (24NSFSC1283), Major projects of scientific and technological innovation 2030 (2021ZD0200801, 2021ZD0200800), National Key Research and Development Program of China (2022YFC2703200, 2016YFC0905000, 2016YFC1200200), Shanghai science and Technology Innovation Fund (21002411100, 20DZ2202000), Shanghai Municipal Science and Technology Major Project (2017SHZDZX01).

### Author contributions

The manuscript was written by all authors. All authors have given approval of the final version of the manuscript. Conceptualization, writing - original draft preparation: SYQ and XYL; Conceptualization: ZXZ and ZLC; Methodology, investigation: XQZ, XLZ, HW, CH and JHZ; Data curation, formal analysis: YXL, FY, YTZ, YZW, QGX, RYC and WB; Data curation, formal analysis, MLY, ZKY and ZYC; Writing - review and editing, project administration, supervision: SYQ and ZXZ.

### Data availability

The RNA-seq (miRNA, circRNA and mRNA & lncRNA) datasets of 18 samples have been deposited in NCBI Gene Expression Omnibus (GEO) with accession ids GSE269011, GSE269012 and GSE269013.

### Conflict of interest

The authors declare that they have no conflict of interest.

### Artificial Intelligence (AI) - assisted technology

No artificial intelligence tools were used in the preparation, writing, editing, or analysis of this manuscript.

## Supplementary Material

Supplementary information

Supplementary data

## Figures and Tables

**Figure 1:**
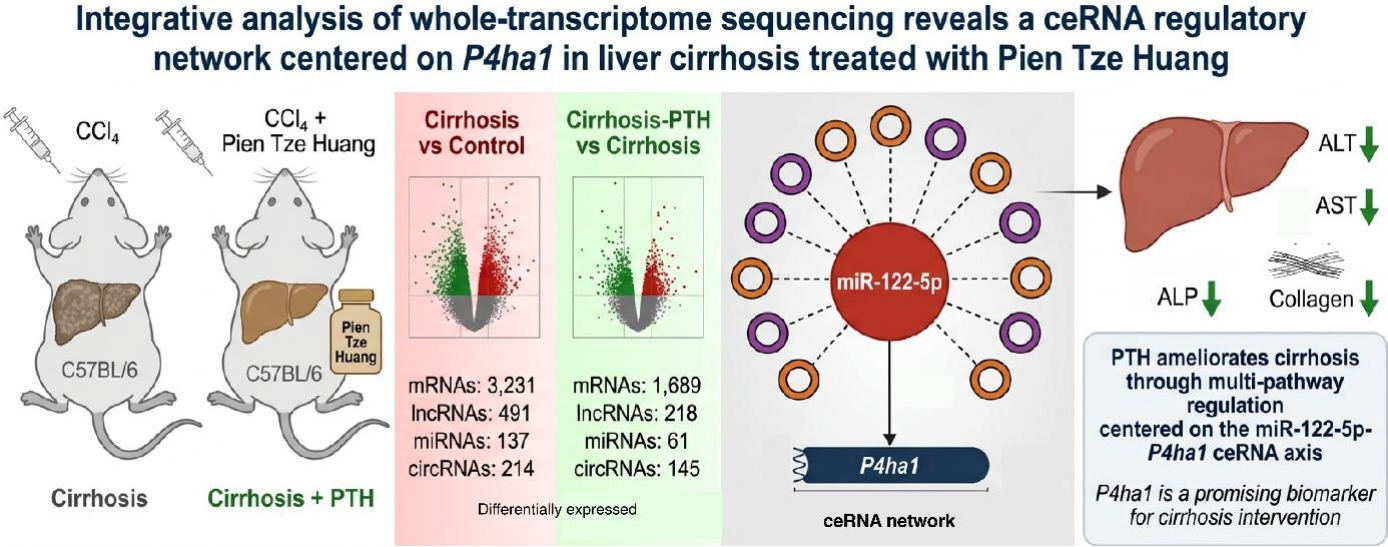
Graphical abstract

## References

[1] Acharya C, Sahingur SE, Bajaj JS (2017). Microbiota, cirrhosis, and the emerging oral-gut-liver axis. JCI Insight.

[2] Bandiera S, Pfeffer S, Baumert TF, Zeisel MB (2015). miR-122--a key factor and therapeutic target in liver disease. J Hepatol.

[3] Bernardo A, St Amand P, Le HQ, Su Z, Bai G (2020). Multiplex restriction amplicon sequencing: a novel next-generation sequencing-based marker platform for high-throughput genotyping. Plant Biotechnol J.

[4] Brown J, Pirrung M, McCue LA (2017). FQC Dashboard: integrates FastQC results into a web-based, interactive, and extensible FASTQ quality control tool. Bioinformatics.

[5] Cao T, Li Q, Huang Y, Li A (2023). plotnineSeqSuite: a Python package for visualizing sequence data using ggplot2 style. BMC Genomics.

[6] Chen L, Shen YH, Wang X, Wang J, Gan Y, Chen N (2006). Human prolyl-4-hydroxylase alpha(I) transcription is mediated by upstream stimulatory factors. J Biol Chem.

[7] Chen Q, Hao H, Guo Z, Zuo Y, Cheng CK, Zhang CL (2024). Pien Tze Huang (PZH) protects endothelial function in diabetic mice. Life Sci.

[8] Chen S, Zhou Y, Chen Y, Gu J (2018). fastp: an ultra-fast all-in-one FASTQ preprocessor. Bioinformatics.

[9] Chen X, Ji ZL, Chen YZ (2002). TTD: Therapeutic Target Database. Nucleic Acids Res.

[10] Chen X, Zhou H, Liu YB, Wang JF, Li H, Ung CY (2006). Database of traditional Chinese medicine and its application to studies of mechanism and to prescription validation. Br J Pharmacol.

[11] Chen Z (2021). Pien Tze Huang (PZH) as a Multifunction Medicinal Agent in Traditional Chinese Medicine (TCM): a review on cellular, molecular and physiological mechanisms. Cancer Cell Int.

[12] DeOcesano-Pereira C, Machado RAC, Chudzinski-Tavassi AM, Sogayar MC (2020). Emerging Roles and Potential Applications of Non-Coding RNAs in Glioblastoma. Int J Mol Sci.

[13] Di Z, Muyun W, Luan C, Hao WU, Ting W, Zhiruo Z (2022). Drug response biomarkers of Pien Tze Huang treatment for hepatic fibrosis induced by carbon tetrachloride. J Tradit Chin Med.

[14] Du TY, Gao YX, Zheng YS (2023). Identification of key genes related to immune infiltration in cirrhosis via bioinformatics analysis. Sci Rep.

[15] Enright AJ, John B, Gaul U, Tuschl T, Sander C, Marks DS (2003). MicroRNA targets in Drosophila. Genome Biol.

[16] Friedländer MR, Mackowiak SD, Li N, Chen W, Rajewsky N (2012). miRDeep2 accurately identifies known and hundreds of novel microRNA genes in seven animal clades. Nucleic Acids Res.

[17] Gaujoux R, Seoighe C (2010). A flexible R package for nonnegative matrix factorization. BMC Bioinformatics.

[18] Gou H, Su H, Liu D, Wong CC, Shang H, Fang Y (2023). Traditional Medicine Pien Tze Huang Suppresses Colorectal Tumorigenesis Through Restoring Gut Microbiota and Metabolites. Gastroenterology.

[19] Hu J, Xu Y, Hao J, Wang S, Li C, Meng S (2012). MiR-122 in hepatic function and liver diseases. Protein Cell.

[20] Huang DQ, Terrault NA, Tacke F, Gluud LL, Arrese M, Bugianesi E (2023). Global epidemiology of cirrhosis - aetiology, trends and predictions. Nat Rev Gastroenterol Hepatol.

[21] Jiang L, Wang M, Lin S, Jian R, Li X, Chan J (2020). A Quantitative Proteome Map of the Human Body. Cell.

[22] Juanola A, Pose E, Ginès P (2025). Liver Cirrhosis: ancient disease, new challenge. Med Clin (Barc).

[23] Kechin A, Boyarskikh U, Kel A, Filipenko M (2017). cutPrimers: A New Tool for Accurate Cutting of Primers from Reads of Targeted Next Generation Sequencing. J Comput Biol.

[24] Kim D, Paggi JM, Park C, Bennett C, Salzberg SL (2019). Graph-based genome alignment and genotyping with HISAT2 and HISAT-genotype. Nat Biotechnol.

[25] Lamb J (2007). The Connectivity Map: a new tool for biomedical research. Nat Rev Cancer.

[26] Langdon WB (2015). Performance of genetic programming optimised Bowtie2 on genome comparison and analytic testing (GCAT) benchmarks. BioData Min.

[27] Langmead B, Salzberg SL (2012). Fast gapped-read alignment with Bowtie 2. Nat Methods.

[28] Li J, Ghazwani M, Zhang Y, Lu J, Li J, Fan J (2013). miR-122 regulates collagen production via targeting hepatic stellate cells and suppressing P4HA1 expression. J Hepatol.

[29] Li Q, Pan X, Li N, Gong W, Chen Y, Yuan X (2021). Identification of Circular RNAs in Hypothalamus of Gilts during the Onset of Puberty. Genes (Basel).

[30] Liu M, Zhou B, Li S, Wang Y, Zheng L, Xiong Y (2026). Pien-Tze-Huang ameliorates autoimmune hepatitis by promoting hepatocyte autophagy via inhibiting miR-342-3p/mTOR signal. J Ethnopharmacol.

[31] Luthra R, Patel KP, Routbort MJ, Broaddus RR, Yau J, Simien C (2017). A Targeted High-Throughput Next-Generation Sequencing Panel for Clinical Screening of Mutations, Gene Amplifications, and Fusions in Solid Tumors. J Mol Diagn.

[32] Melis M, Marino R, Tian J, Johnson C, Sethi R, Oertel M (2024). Mechanism and Effect of HNF4α Decrease in a Rat Model of Cirrhosis and Liver Failure. Cell Mol Gastroenterol Hepatol.

[33] Pertea M, Pertea GM, Antonescu CM, Chang TC, Mendell JT, Salzberg SL (2015). StringTie enables improved reconstruction of a transcriptome from RNA-seq reads. Nat Biotechnol.

[34] Roderburg C, Urban GW, Bettermann K, Vucur M, Zimmermann H, Schmidt S (2011). Micro-RNA profiling reveals a role for miR-29 in human and murine liver fibrosis. Hepatology.

[35] Sadowska A, Osiński P, Roztocka A, Kaczmarz-Chojnacka K, Zapora E, Sawicka D (2023). Statins-From Fungi to Pharmacy. Int J Mol Sci.

[36] Sakaida I, Matsumura Y, Kubota M, Kayano K, Takenaka K, Okita K (1996). The prolyl 4-hydroxylase inhibitor HOE 077 prevents activation of Ito cells, reducing procollagen gene expression in rat liver fibrosis induced by choline-deficient L-amino acid-defined diet. Hepatology.

[37] Shi Y, Yang F, Wei S, Xu G (2017). Identification of Key Genes Affecting Results of Hyperthermia in Osteosarcoma Based on Integrative ChIP-Seq/TargetScan Analysis. Med Sci Monit.

[38] Sporea I, Raţiu I, Bota S, Şirli R, Jurchiş A (2013). Are different cut-off values of liver stiffness assessed by transient elastography according to the etiology of liver cirrhosis for predicting significant esophageal varices?. Med Ultrason.

[39] Sun K (2020). Ktrim: an extra-fast and accurate adapter- and quality-trimmer for sequencing data. Bioinformatics.

[40] Toden S, Zumwalt TJ, Goel A (2021). Non-coding RNAs and potential therapeutic targeting in cancer. Biochim Biophys Acta Rev Cancer.

[41] Turco L, Reiberger T, Vitale G, La Mura V (2023). Carvedilol as the new non-selective beta-blocker of choice in patients with cirrhosis and portal hypertension. Liver Int.

[42] Varet H, Brillet-Guéguen L, Coppée JY, Dillies MA (2016). SARTools: A DESeq2- and EdgeR-Based R Pipeline for Comprehensive Differential Analysis of RNA-Seq Data. PLoS One.

[43] Vitiello M, Evangelista M, Zhang Y, Salmena L, Pandolfi PP, Poliseno L (2020). PTENP1 is a ceRNA for PTEN: it's CRISPR clear. J Hematol Oncol.

[44] Wang H, Chen F, Wang S, Li Y, Liu T, Li Y (2023). Evaluation and mechanism study of Pien Tze Huang against EV-A71 infection. Front Pharmacol.

[45] Wang T, Zhu J, Gao L, Wei M, Zhang D, Chen L (2022). Identification of circular RNA biomarkers for Pien Tze Huang treatment of CCl4‑induced liver fibrosis using RNA‑sequencing. Molecular Medicine Reports.

[46] Wen J, Friedman JR (2012). miR-122 regulates hepatic lipid metabolism and tumor suppression. J Clin Invest.

[47] Wu K, Lin F (2024). Lipid Metabolism as a Potential Target of Liver Cancer. J Hepatocell Carcinoma.

[48] Wu Q, He Y, Liu X, Luo F, Jiang Y, Xiang M (2023). Cancer stem cell-like cells-derived exosomal lncRNA CDKN2B-AS1 promotes biological characteristics in thyroid cancer via miR-122-5p/P4HA1 axis. Regen Ther.

[49] Wu T, Hu E, Xu S, Chen M, Guo P, Dai Z (2021). clusterProfiler 4.0: A universal enrichment tool for interpreting omics data. Innovation (Camb).

[50] Zeng C, Wang YL, Xie C, Sang Y, Li TJ, Zhang M (2015). Identification of a novel TGF-β-miR-122-fibronectin 1/serum response factor signaling cascade and its implication in hepatic fibrogenesis. Oncotarget.

[51] Zhang F, Pei S, Xiao M (2024). Identification of functional genes in liver fibrosis based on bioinformatics analysis of a lncRNA-mediated ceRNA network. BMC Med Genomics.

[52] Zhang HD, Jiang LH, Hou JC, Zhou SY, Zhong SL, Zhu LP (2018). Circular RNA hsa_circ_0072995 promotes breast cancer cell migration and invasion through sponge for miR-30c-2-3p. Epigenomics.

[53] Zhao J, Hu H, Wan Y, Zhang Y, Zheng L, Hong Z (2017). Pien Tze Huang Gan Bao ameliorates carbon tetrachloride-induced hepatic injury, oxidative stress and inflammation in rats. Exp Ther Med.

[54] Zheng S, Xue T, Wang Q, Zhang P, Qi W, Xue C (2025). Chinese Medicine for the Treatment of Liver Cirrhosis: The Mechanism of Cellular Autophagy. Am J Chin Med.

[55] Zhu J, Zhang D, Wang T, Chen Z, Chen L, Wu H (2021). Target identification of hepatic fibrosis using Pien Tze Huang based on mRNA and lncRNA. Sci Rep.

