# Supplementary material for: Integrative analysis of whole-transcriptome sequencing reveals a ceRNA regulatory network centered on P4ha1 in liver cirrhosis treated with Pien Tze Huang
Source: EXCLI J. 2026 Jul 13;25:1070–89. doi: 10.17179/excli2026-9413 (PMC13402739; doi:10.17179/excli2026-9413)
Supplement: Supplementary information [file EXCLI-25-1070-s-001.pdf]

EXCLI Journal 2026;25:1070-1089 – ISSN 1611-2156
Received: March 14, 2026, accepted: June 03, 2026, published: July 13, 2026

Supplementary information to:

Original article:

INTEGRATIVE ANALYSIS OF WHOLE-TRANSCRIPTOME SEQUENCING REVEALS A CERNA REGULATORY NETWORK CENTERED ON P4ha1 IN LIVER CIRRHOSIS TREATED WITH PIEN TZE HUANG

Yunxiao Lin[1#], Fan Yang[1#], Yingtian Zhang[1#], Xiaoqin Zhang[2#], Xiangyi Li[1], Zhiliang Chen[2], Xianglong Zhao[1], Yongzhi Wang[1], Hao Wu[1], Cong Huai[1], Qiange Xiao[1], Wei Bao[1], Minglei Yang[3], Ruoyu Chen[1], Zhongyu Cao[1], Jinhang Zhu[1], Zekun Yu[1], Zexiu Zhang[2*], Shengying Qin[1*]

[1] Bio-X Institutes, Key Laboratory for the Genetics of Developmental and Neuropsychiatric Disorders (Ministry of Education), Shanghai Jiao Tong University, Shanghai, 200030, China
[2] Fujian Provincial Key Laboratory of Pien Tze Huang Natural Medicine Research and Development, Zhangzhou Pien Tze Huang Pharmaceutical Co., Ltd, Zhangzhou, 363000, China
[3] Department of Pathology, The First Affiliated Hospital of Zhengzhou University, Zhengzhou, 450052, China

[#] These authors have contributed equally as first authors.

* **Corresponding authors:** Professor Shengying Qin, Bio-X Institutes, Key Laboratory for the Genetics of Developmental and Neuropsychiatric Disorders (Ministry of Education), Shanghai Jiao Tong University, 1954 Hua Shan Road, Shanghai 200030, P.R. China; E-mail: chinsir@sjtu.edu.cn; Tel.: 021-62932779
Professor Zexiu Zhang, Fujian Provincial Key Laboratory of Pien Tze Huang Natural Medicine Research and Development, Zhangzhou Pien Tze Huang Pharmaceutical Co., Ltd., 50-1 Xinhua North Road, Xiangcheng, Zhangzhou, Fujian 363000, P.R. China; E-mail: zzx@zzpzh.com; Tel.: 0596-2305448

https://dx.doi.org/10.17179/excli2026-9413

This is an Open Access article distributed under the terms of the Creative Commons Attribution License (https://creativecommons.org/licenses/by/4.0/).

EXCLI Journal 2026;25:1070-1089 – ISSN 1611-2156
Received: March 14, 2026, accepted: June 03, 2026, published: July 13, 2026

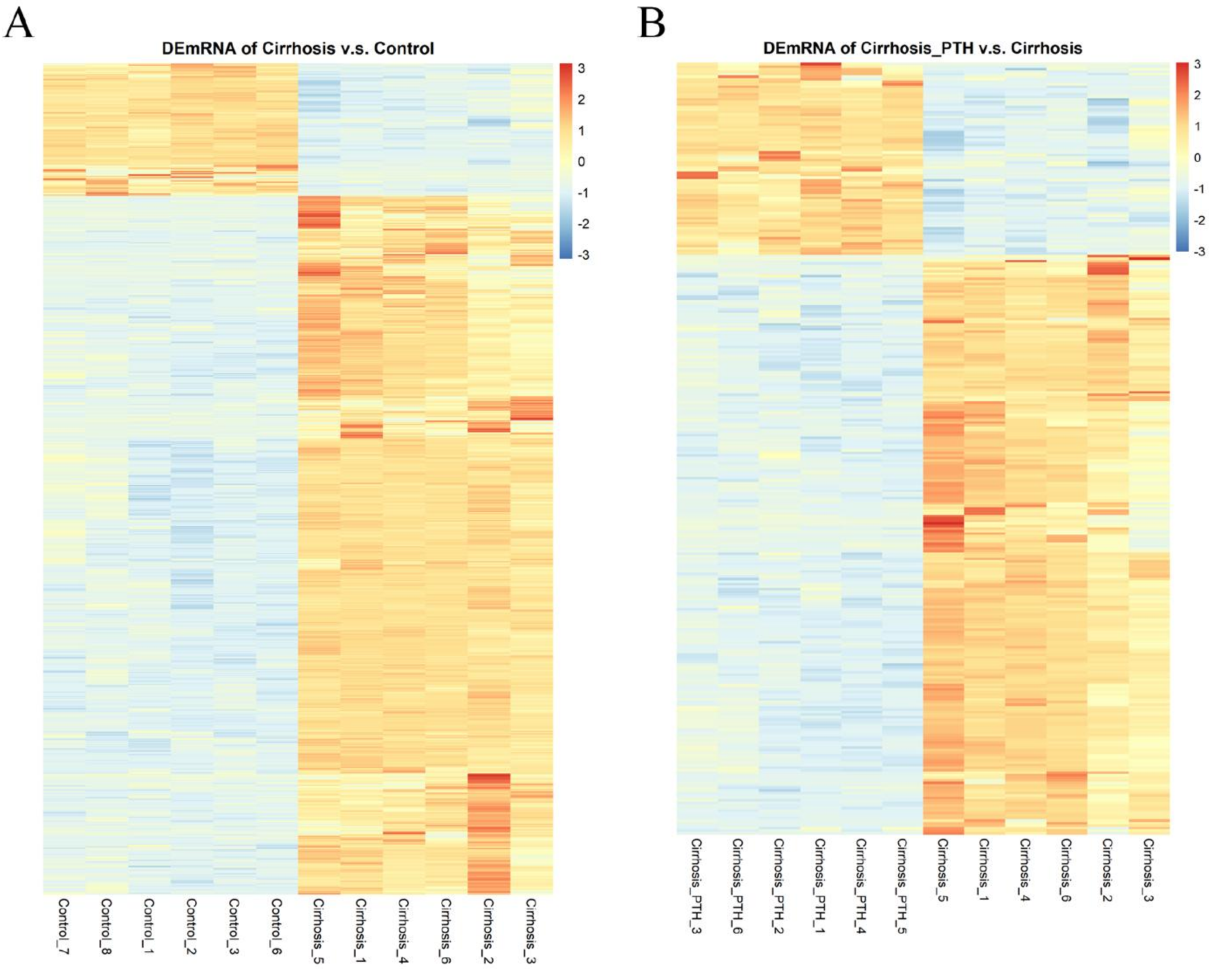

Supplementary Figure 1: Differential expression mRNA for Cirrhosis vs. Control and Cirrhosis-PTH vs. Cirrhosis comparison group

Received: March 14, 2026, accepted: June 03, 2026, published: July 13, 2026

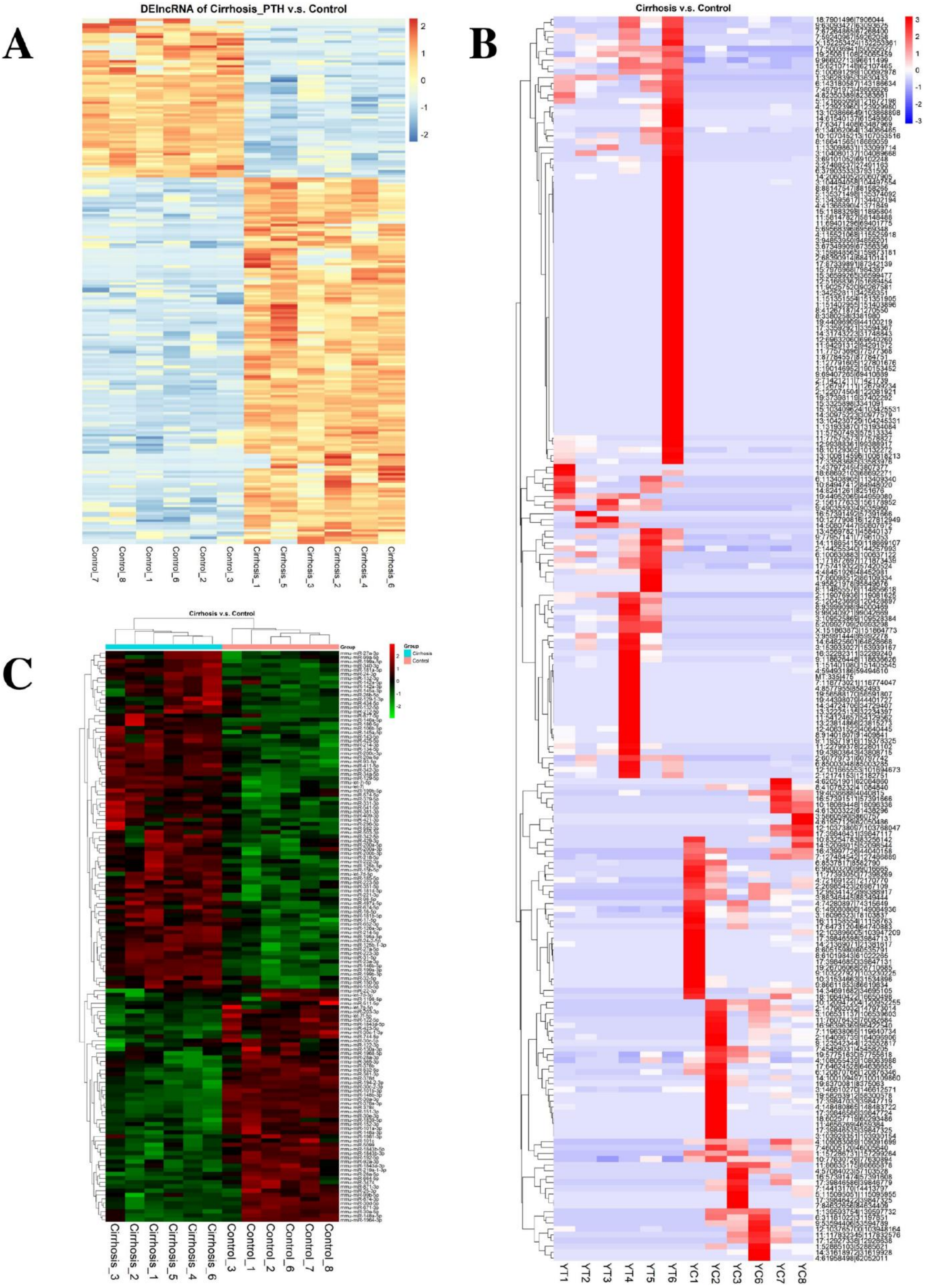

Supplementary Figure 2: Differential expression analysis for lncRNA, miRNA and circRNA in the Cirrhosis vs. Control groups

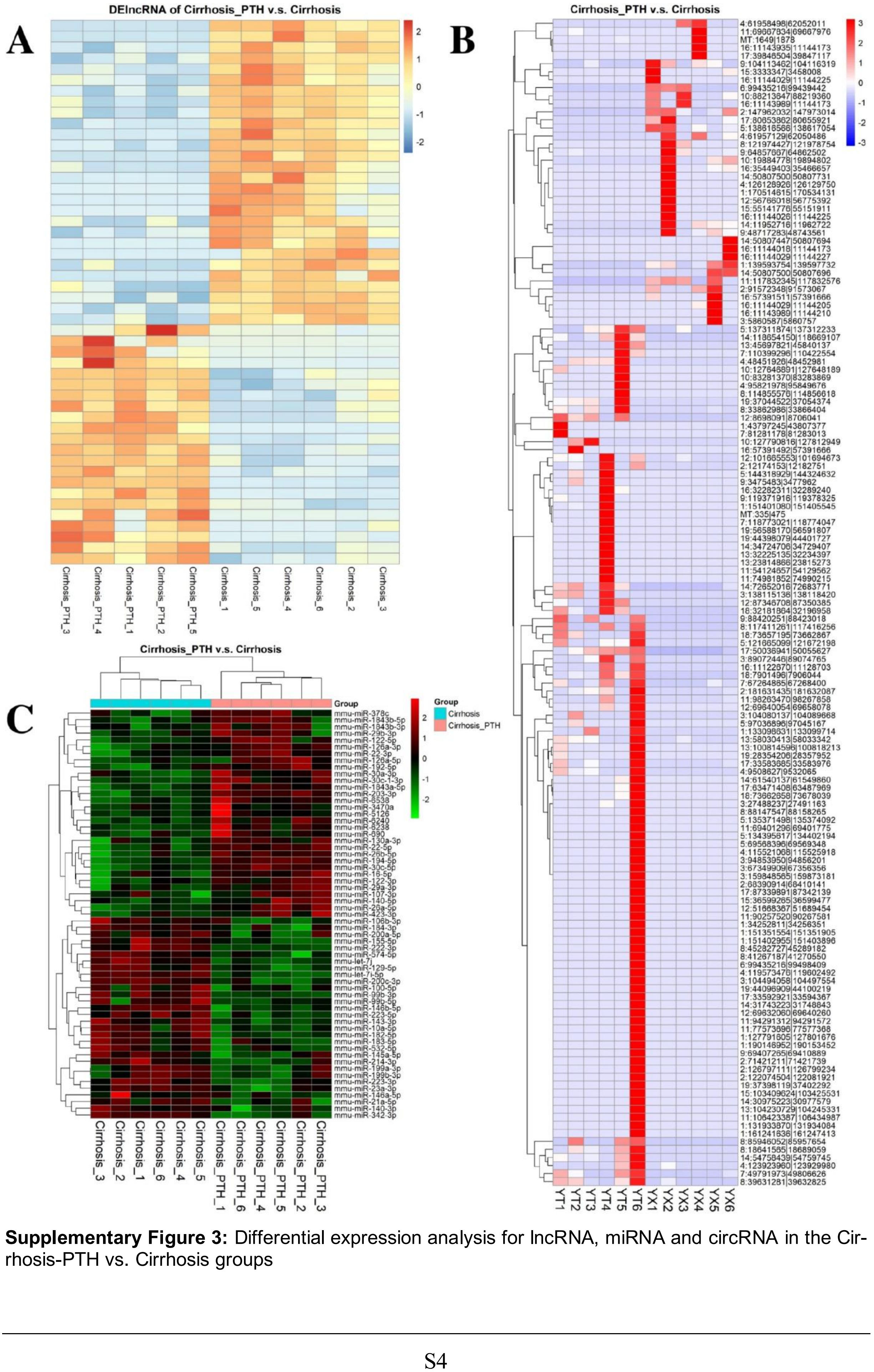

Supplementary Figure 3: Differential expression analysis for lncRNA, miRNA and circRNA in the Cirrhosis-PTH vs. Cirrhosis groups

EXCLI Journal 2026;25:1070-1089 – ISSN 1611-2156
Received: March 14, 2026, accepted: June 03, 2026, published: July 13, 2026

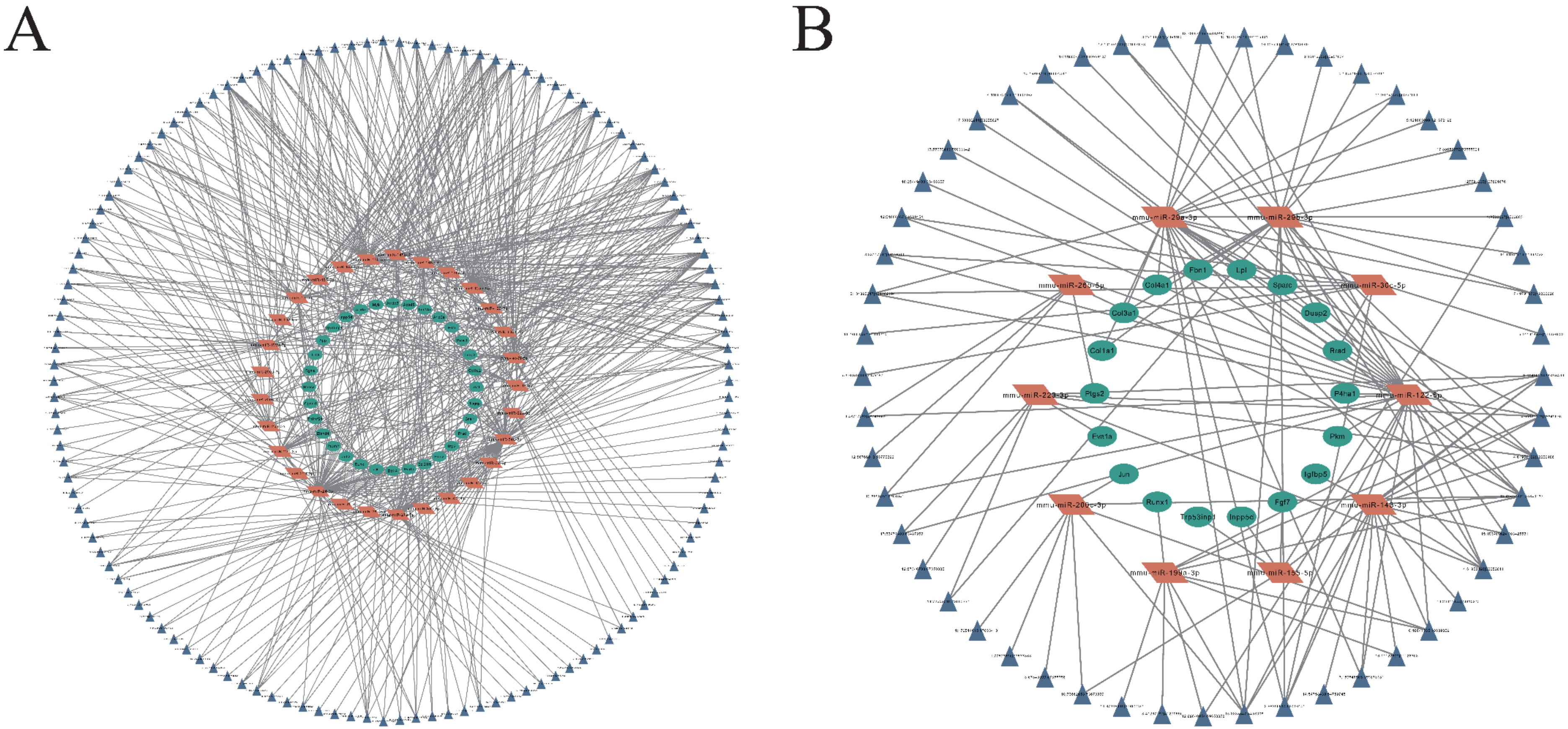

Supplementary Figure 4: Construction of the ceRNA Regulatory Network: (**A**)The ceRNA regulatory network for the Cirrhosis vs. Control groups; (**B**) The ceRNA regulatory network for the Cirrhosis-PTH vs. Cirrhosis groups. CircRNA, miRNA, and mRNA were indicated by equilateral triangular, circular diamond, respectively

EXCLI Journal 2026;25:1070-1089 – ISSN 1611-2156
Received: March 14, 2026, accepted: June 03, 2026, published: July 13, 2026

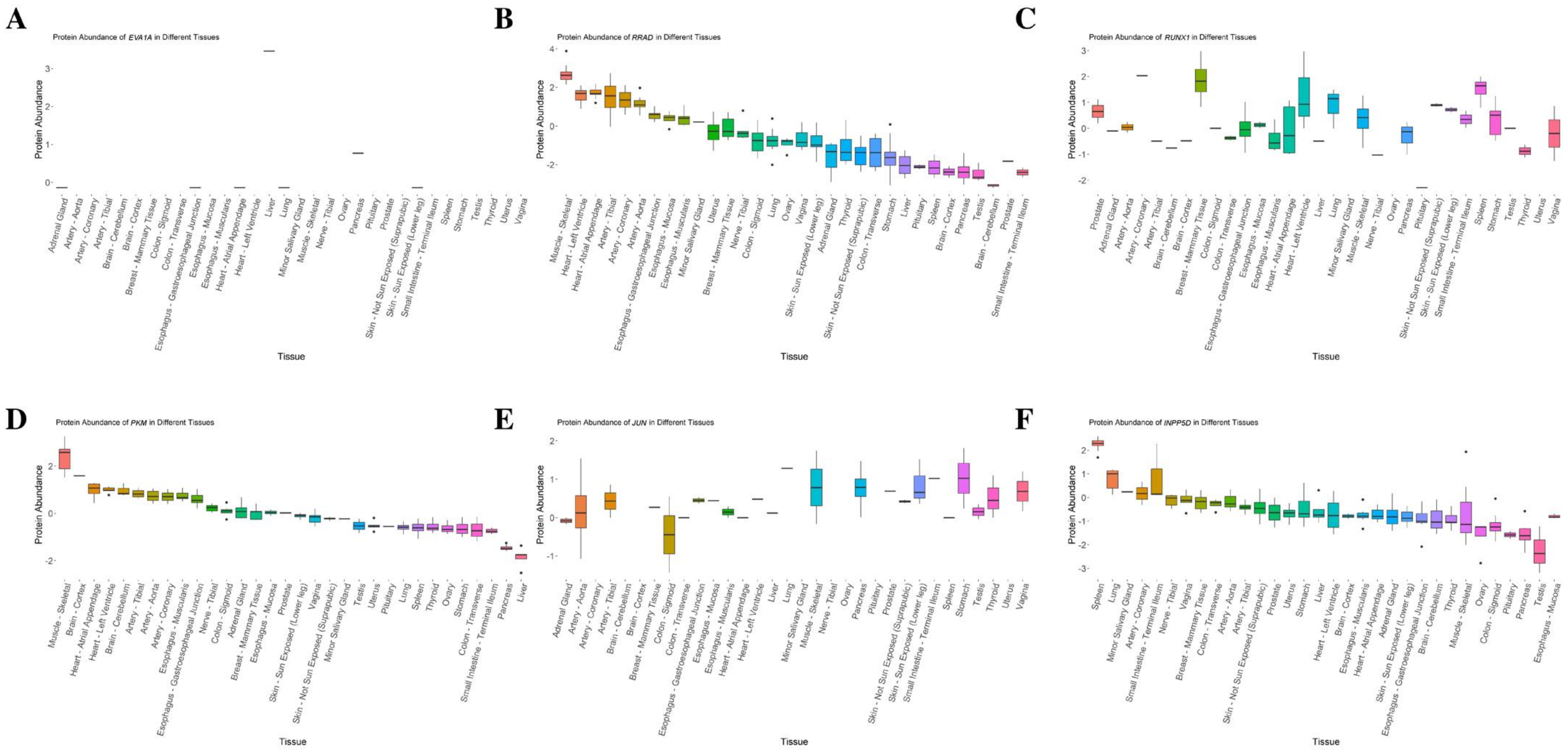

Supplementary Figure 5: Protein abundance of *EVA1A, RRAD, RUNX1, PKM, JUN* and *INPP5D* in different tissues from the TTD database

EXCLI Journal 2026;25:1070-1089 – ISSN 1611-2156
Received: March 14, 2026, accepted: June 03, 2026, published: July 13, 2026

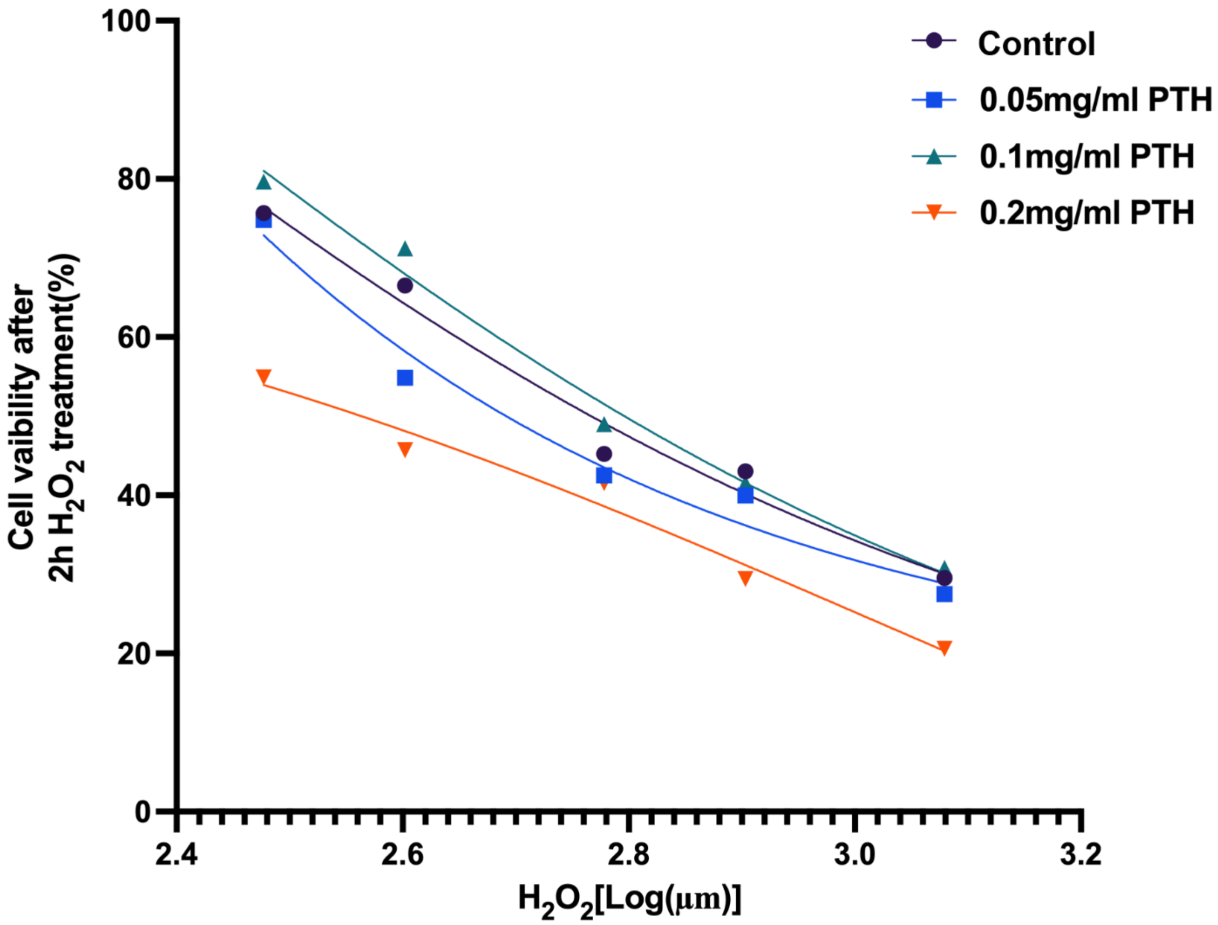

Supplementary Figure 6: Dose-response optimization of PTH and H_2O_2 in HepG2 cells: Cells were pretreated with indicated concentrations of PTH (0.05, 0.1, or 0.2 mg/mL) for 24 h, followed by exposure to increasing concentrations of H_2O_2 (0–1000 µM) for 2 h. Cell viability was then measured by CCK-8 assay. Data are presented as mean ± SEM (n = 3 independent experiments).
